# Analysing fungal microbiome differences between the roots of healthy and diseased Chinese hickory (*Carya cathayensis*) trees

**DOI:** 10.1038/s41598-025-27645-y

**Published:** 2025-12-17

**Authors:** Shengrong Su, Genshan Li, Shiqi Ge, Hong-Hui Wang, Xiao-Fang Pan, Qi Yao, Xiufeng Cao, Miao Zhang, Ai-juan Hong, Lishan Fang, Dacai Zhai, Xiao-Hui Bai

**Affiliations:** 1 College of Life and Environment Science, Huangshan University, Huangshan, 245041 Anhui People’s Republic of China; 2 School of Food and Biological Engineering, Hefei University of Technology, Hefei, 230009 Anhui People’s Republic of China; 3 Huangshan Institute of Product Quality Inspection, Huangshan, 245000 Anhui People’s Republic of China; 4 Forestry Science and Technology Promotion Center of Shexian, Huangshan, 245200 Anhui People’s Republic of China; 5 Huangshan Shanye Local Specialty Co., LTD., Huangshan, 245200 Anhui People’s Republic of China; 6 Huangshan Tianzhiyuan Agricultural Products Co., LTD., Huangshan, 245213 Anhui People’s Republic of China

**Keywords:** Chinese hickory (*Carya cathayensis*), Rhizosphere soil, Root tissue, Bulk soil, Fungal communities, Microbial communities, Environmental microbiology, Fungi

## Abstract

Chinese hickory (*Carya cathayensis*), an important economic nut species in China, has recently suffered significant losses due to root rot. Previous *16S rRNA* amplicon sequencing suggested that the bacterial dysbiosis may contribute to root rot, but the specific pathogens remained unclear. In this study, fungal community analysis revealed that Ascomycota and Basidiomycota dominated the rhizosphere soil, bulk soil, and root tissues, accounting for approximately 93.63% of total fungal communities. The relative abundance of Basidiomycota were more abundant in healthy root tissues, whereas the relative abundance of Ascomycota were enriched in diseased and dead roots. Interestingly, at the genus level, the dominant fungi *Xylaria* and *Ilyonectria* were detected exclusively in diseased and dead trees, while *Condinaea* and *Gliocladiopsis* were primarily found in dead trees. These genera have been previously reported as root rot pathogens in various plants, suggesting their association with *C. cathayensis* root rot. Notably, two biocontrol fungi, *Chaetomium* and *Trichoderma*, were also present in diseased and dead trees, highlighting potential strategies for disease management. Overall, this study identifies for the first time the potential pathogenic fungi responsible for *C. cathayensis* root rot and highlights candidate biocontrol agents, providing a foundation for future disease verification and control efforts.

**Supplementary Information:**

The online version contains supplementary material available at 10.1038/s41598-025-27645-y.

## Introduction

Chinese hickory (*Carya cathayensis*) is primarily distributed in Shexian and Ningguo in Anhui Province, as well as in Lin’an and Chun’an in Zhejiang Province^1^. The hickory kernel, a key product of the hickory tree, is prized for its high oil content, significant protein levels, and essential minerals that support human health^2^. Due to its unique flavour and excellent nutritional value, the hickory kernel is highly popular among consumers and holds substantial economic value^3^. As a result, the Chinese hickory industry has become a significant economic contributor in these regions. However, the increasing economic value of Chinese hickory has led to overreliance on chemical fertilizers and herbicides by farmers seeking higher yields. This has resulted in nutrient imbalances in the soil, increased soil acidification, and a decline in biodiversity within Chinese hickory forests. Consequently, the ecosystem of these forests has degraded, negatively impacting the broader regional forest ecosystem.

Recently, an unknown root rot disease has emerged in Chinese hickory forests, exhibiting typical soil-borne characteristics. The pathogen invades through the fine roots and progressively spreads to the lateral and main roots^4–6^. Affected mature trees exhibit symptoms such as chlorosis, yellowing leaves, reduced leaf size, and premature defoliation. Under high temperatures and drought conditions during the summer, the leaves tend to wilt. Anatomical analysis of the diseased roots reveals that the fine root cortex loses its luster and can be easily peeled off, while the xylem darkens and turns yellow. As the disease progresses, root rot intensifies, eventually leading to the death of the entire tree^5^. This root rot has spread widely across major hickory kernel production areas, with some regions reporting an incidence rate as high as 90%^6^. The disease has caused substantial tree mortality, resulting in significant economic losses for farmers and posing a serious threat to the sustainability of the Chinese hickory industry and regional ecological stability. Thus, it is critical to identify the underlying cause of *C. cathayensis* tree root rot and develop efficient control strategies.

The composition of pathogens responsible for root rot varies significantly by region^7^. For instance, in Heilongjiang Province, *Fusarium oxysporum* is the primary pathogen causing soybean root rot^8^, while in Sichuan Province, the main isolates of soybean root rot include *F. oxysporum*, *F. graminearum*, and *F. equiseti*^9^. In North America, including Canada, *F. oxysporum* and *F. solani* are the predominant pathogens^10^. These regional variations in dominant pathogens may help explain why targeted chemical treatments often yield limited results, despite *F. oxysporum* being frequently reported as the causative agent of *C. cathayensis* root rot^11^. Additionally, several studies have emphasized that the role of imbalances in soil microbial communities in the development of root rot disease^12–15^. Therefore, understanding the differences in microbial community composition between the soil and root tissues of healthy and diseased *C. cathayensis* trees is crucial for developing more effective disease control strategies.

Root rot, caused by bacteria, viruses, oomycetes, and fungi, is one of the most significant plant diseases worldwide^16,17^. Previous studies utilized high-throughput sequencing to analyze microbial composition differences in the rhizosphere of *C. cathayensis* trees, revealing disruptions in the balanced microbial state under various health conditions^5^. However, the specific composition of the fungal community and its dynamic changes during the transition from health to disease remain unclear. In this study, we systematically collected root tissue, rhizosphere soil, and bulk soil samples from both healthy and diseased *C. cathayensis* trees. Using high-throughput sequencing, we analyzed the structure and dynamics of the fungal community to: (1) identify characteristic differences in fungal communities between healthy and diseased trees; (2) provide a foundation for isolating and identifying potential pathogenic fungi; and (3) inform the development of ecological control strategies for root rot disease.

## Materials and methods

### Experimental design and sample collection

To reveal the difference in microbial community composition of *C. cathayensis* trees with varying health status, we divided the trees within the same plantations into three groups: dead plants (DP group), diseased plants (SP group), and healthy plants (NP group). *C. cathayensis* trees with heights ranging from 7.5 to 8.5 m and spaced 30–50 m apart were randomly selected as samples, based on criteria from the previous study^5^. Rhizosphere soil (RS), bulk soil (BS), and root tissues (RT) were collected from the DP, SP, and NP groups of *C. cathayensis* trees as described previously^18^. The fungal community composition of these samples was determined using ITS high-throughput sequencing, enabling a precise comparison of microbial community differences across the DP, SP, and NP groups. Detailed protocols for sample collection and processing can be found in our previous report^5^.

### Extraction of the microbial genomic DNA

Microbial genomic DNA was extracted from root tissues and soil using CTAB method, as previously described^19^. Briefly, 0.5 g of root tissues or soil was treated with 1 ml of PBS buffer (pH 7.4, 10 mM Na_2_HPO_4_, 1.8 mM KH_2_PO_4_, 2.7 mM KCl, and 200 mM NaCl) and ground thoroughly with liquid nitrogen. Subsequently, 1 ml of CTAB lysate and 20 µl 10 mg/ml lysozyme were added, and the mixture was incubated in a water bath at 65 °C for 3 h. After centrifugation at 12, 000 rpm for 10 min, the suspension was treated sequentially with an equal volume of phenol: chloroform: isoamyl alcohol (25:24:1) and chloroform: isoamyl alcohol (24:1). The genomic DNA was then precipitated by adding 3/4 volume of ice-cold isopropyl alcohol. Finally, the DNA precipitate was washed three times with 75% ethanol and then resuspended with 60 µl of elution buffer (10 mM Tris-HCl pH 8.5, 0.2 mM EDTA).

### Internal transcribed spacer 1 region amplification, quantitation, and sequencing

The internal transcribed spacer 1 (ITS1) region of each sample was amplified for sequencing using the primer pair (ITS5: 5´-GGAAGTAAAAGTCGTAACAAGG-3´, ITS2: GCTGCGTTCTTCATCGATGC-3´) containing a barcode^20^. PCR amplification was performed using the Phusion High-Fidelity PCR Master Mix kit (New England Biolabs, USA) according to the manufacturer’s instructions. Amplified products were detected by 2% agarose gel electrophoresis. Samples with bands between 200 and 400 bp were extracted using the QIAquick Gel Extraction Kit (Qiagen, Germany) for sequencing. The library was then constructed using the TruSeq DNA PCR-Free Sample Preparation Kit (Illumina, USA). After quantitative analysis, the constructed library was sequenced on the Illumina HiSeq2500 platform.

### Sequence processing

Raw data obtained from the HiSeq2500 runs were processed using the the EasyAmplicon pipeline^21^ with default settings, based on the barcode sequences. High-quality filtering was applied to remove reads with ambiguous or homologous sequences, as well as those shorter than 200 bp, from the raw tags in order to obtain clean tags. Taxonomic identification of fungal amplicon sequence variants (ASVs) was performed using the UNITE database^22^. Unique sequences were processed using unoise3^23^ with a minimum size of 10 to reduce the dataset size and achieve single-base ASV accuracy. Meanwhile, sequences assigned to chloroplasts and mitochondria were excluded. The EasyAmplicon pipeline^21^ was used to evaluate richness and diversity indices including ACE, Chao1, Richness, Simpson, and Shannon. Additionally, the pipeline generated feature tables and phylogenetic trees.

### Statistical analysis

Statistical analysis was performed using R software (version 4.2.2), as previously described^5^. Briefly, ANOVA with Tukey HSD was used to compare the statistical significance of the α-diversity between different groups (*P* < 0.05). β-diversity was performed using the permutational multivariate analysis of variance (PERMANOVA). The edgeR package^24^ was employed to compare the differences in ASV abundance between different groups, with the Benjamini–Hochberg method applied to control the false discovery rate.

Network analysis and visualization were performed using the ggClusterNet package (version 0.1.73)^25^, with a correlation coefficient (R) greater than 0.8 and a *P*-value less than 0.05. ASVs with a relative abundance > 0.01% were selected for network relationship exploration, and the topological features of the network were assessed. The network graph was color-coded according to modules, and network vulnerability was calculated using the ggClusterNet package, with default thresholds of *R* > 0.8 and *P* < 0.05. For cross-network analysis between bacteria and fungi, the corBionetwork function was used, with the same thresholds of *R* > 0.8 and *P* < 0.05.

## Results

### Sequencing data characterisation

A total of 1,695,573 ITS1 amplicon sequence reads were obtained from 27 samples, with an average of 62,799 reads per sample. After quality control using the EasyAmplicon pipeline, an average of 61,425 clean reads were retained. The sequences were taxonomically classified into fungal ASVs based on the UNITE database, resulting in 879 ASVs for RT, 978 ASVs for RS, and 996 ASVs for BS, respectively. Six fungal ASVs were identified as contaminants using MicroDecon^26^ and were removed from the dataset. Sequentially, the rarefaction curves were drawn to analyze the sequencing depth. Our results showed that as the sequencing depth gradually deepened, the species richness curve gradually flattened, indicating that the sequencing depth could truly reflect the composition of the entire fungal microbiome (Supplementary Fig. 1).

### Alpha diversity

**Fig. 1:**
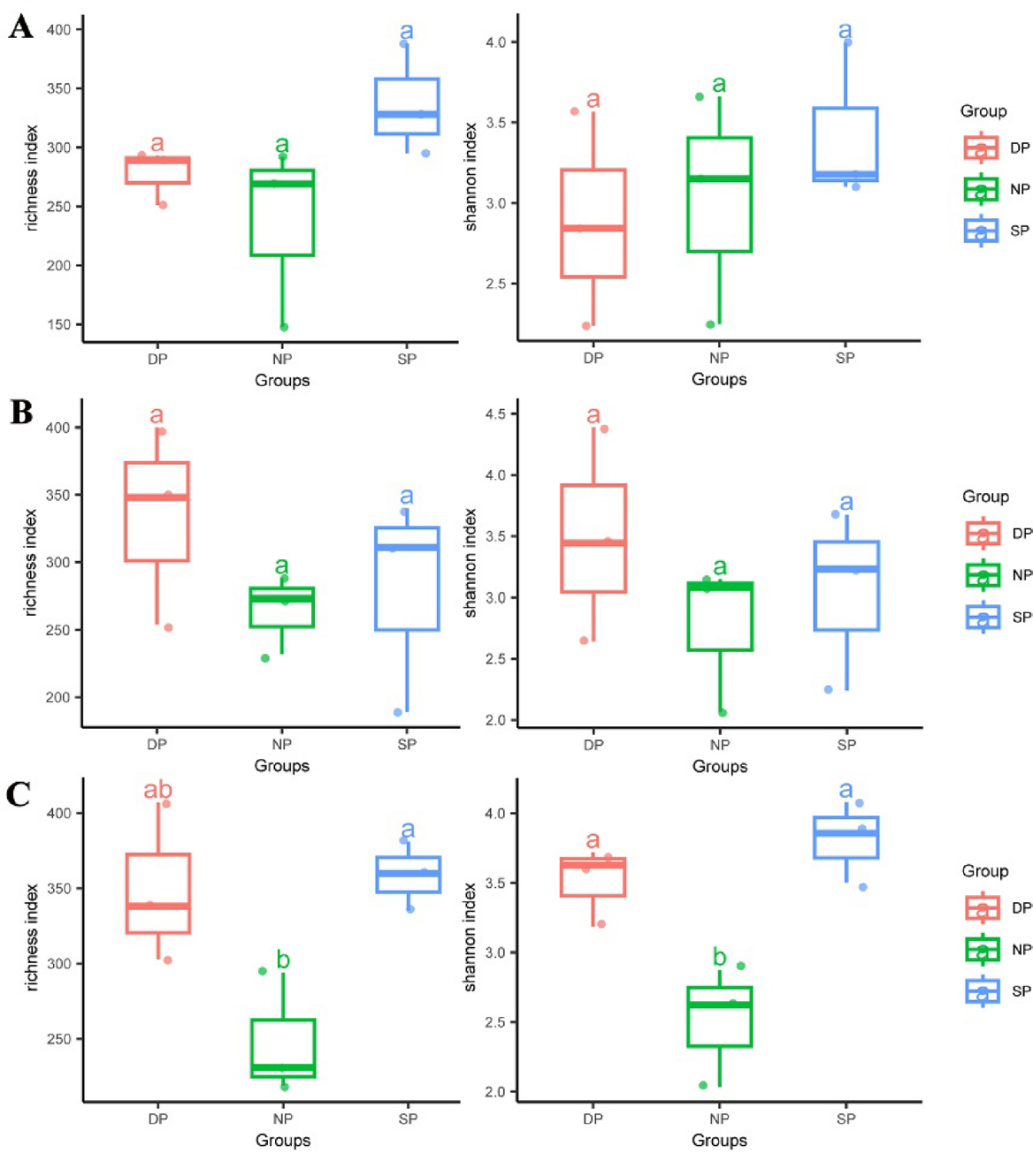
The Richness and Shannon indices in (**A**) RT, (**B**) RS, and (**C**) BS samples. RT, RS, and BS are root tissue, rhizosphere soil, and bulk soil, respectively. DP, NP, and SP represent dead, healthy, and diseased *C. cathayensis* trees, respectively. The first (25%), third (75%) and median quartiles of each data set are shown in the box plots. Significant differences (*P* < 0.05) between datasets are indicated in lowercase.

Species richness and Shannon indices were used to analyze the alpha diversity of RT, RS, and BS in *C. cathayensis* trees. In RT, the species richness indices for the DP, NP, and SP were 278, 236, and 337, respectively (Fig. 1A). The Shannon indices for the DP, NP, and SP were 2.88, 3.02, and 3.42, respectively (Fig. 1A). No significant difference were observed between the species richness and Shannon indices of the DP, NP, and SP groups (Fig. 1A). In RS, both the species richness and Shannon indices followed a same trend. The richness indices for the DP, NP, and SP were 334, 264, and 280, respectively (Fig. 1B), and the Shannon indices were 3.49, 2.76, and 3.05, respectively (Fig. 1B). In BS, the richness indices for the DP, NP, and SP were 349, 248, and 359, respectively (Fig. 1C), and the Shannon indices were 3.51, 2.51, and 3.81, respectively. Significant differences were found between these groups (*P* < 0.05) (Fig. 1C).

### Beta diversity

**Fig. 2:**
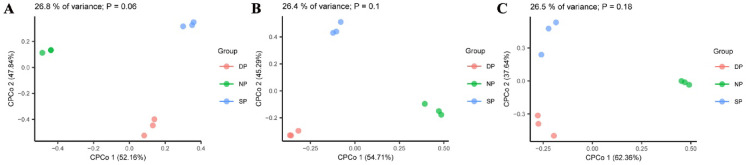
Bray-Curtis distances of (**A**) root tissue, (**B**) rhizosphere soil, and (**C**) bulk soil plotted using constrained PCoA. Each point is coloured by the different group of dead, healthy, and diseased trees. Dead, healthy, and diseased trees are abbreviated DP, NP, and SP, respectively.

Beta diversity of RT, RS, and BS in *C. cathayensis* trees was analyzed using Constrained PCoA based on Bray–Curtis distance. In RT, the results indicated differences in fungal community beta diversity among the DP, NP, and SP groups (*P* = 0.06), explaining 26.8% of the variance (Fig. 2A). In RS, fungal communities also exhibited differences between the DP, NP, and SP groups (*P* = 0.1), accounting for 26.4% of the variation, although the difference was not statistically significant (Fig. 2B). Similarly, differences were observed in fungal communities between the DP, NP, and SP groups of BS (*P* = 0.18), which accounted for 26.5% of the variation (Fig. 2C).

### Composition of the fungal community in NP, SP, and DP groups

**Fig. 3:**
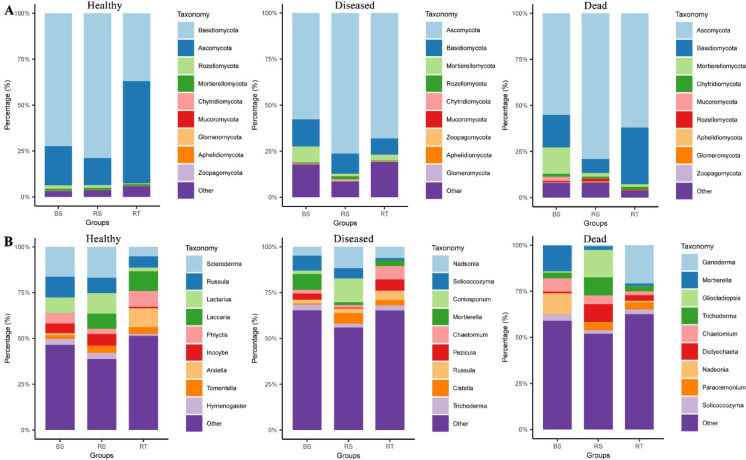
Top 10 dominant fungi relative abundance in different groups of *C. cathayensis* trees. Comparison of the 10 most abundant fungi at the (**A**) phylum level and (**B**) genus level in healthy, diseased, and dead trees. RT, RS, and BS refer to root tissue, rhizosphere soil, and bulk soil, respectively.

The healthy (NP), diseased (SP), and dead (DP) *C. cathayensis* trees were further divided into RT, RS, and BS groups to better analyze differences in fungal communities. The top 10 fungi at the phylum and genus level are shown in Fig. 3. In the NP group, the top 10 abundant fungal phyla across RT, RS, and BS specimens were Basidiomycota (36.97–78.80%), Ascomycota (14.73–55.63%), Rozellomycota (0.37–1.94%), Mortierellomycota (1.05–1.20%), Chytri-diomycota (0.09–0.15%), Mucoromycota (0.00–0.09%), Glomeromycota (0.00–0.09%), Aphelidiomycota (0.01–0.04%), and Zoopagomycota (0.00–0.01%) (Fig. 3A). Although the ranking of the top 10 fungal communities was basically the same across RT, RS, and BS groups, their relative abundances varied significantly. For example, Basidiomycota was most abundant in the RS and BS groups, whereas Ascomycota dominated the RT group. Interestingly, the combined proportion of Basidiomycota and Ascomycota in RT, RS, and BS groups was 92.59,93.53, and 93.63%, respectively, making them the two largest fungal phyla (Fig. 3A). The top 10 fungal genera in the NP group for RT, RS, and BS specimens included *Scleroderma* (5.16–16.83%), *Russula* (6.11–11.33%), *Lactarius* (1.99–11.24%), *Laccaria* (0.11–10.83%), *Phlyctis* (2.76–8.63%), *Inocybe* (0.75–6.51%), *Arxiella* (0.05–10.36%), *Tomentella* (2.04–3.81%), and *Hymenogaster* (1.04–3.34%) (Fig. 3B).

The top 10 fungal phyla in RT, RS, and BS specimens from the SP group were Ascomycota (57.77–76.23%), Basidiomycota (8.76–14.69%), Mortierellomycota (1.42–8.67%), Rozellomycota (0.35–1.62%), Chytri-diomycota (0.39–0.85%), Mucoromycota (0.07–0.25%), Zoopagomycota (0.02–0.19%), Aphelidiomycota (0.06–0.08%), and Glomeromycota (0.03–0.06%) (Fig. 3A). Similarly, the top 10 fungal genera in these samples were *Nadsonia* (4.82–11.67%), *Solicoccozyma* (1.29–8.19%), *Coniosporium* (0.00–13.05%), *Mortierella* (1.41–8.65%), *Chaetomium* (1.42–7.32%), *Pezicula* (0.46–6.18%), *Russula* (2.18–5.07%), *Cistella* (0.55–5.77%), and *Trichoderma* (1.99–3.10%) (Fig. 3B).

In the DP group, the top 10 dominant fungal phyla in RT, RS, and BS specimens were Ascomycota (55.20–79.10%), Basidiomycota (7.55–30.73%), Mortierellomycota (1.40–14.25%), Chytridiomycota (0.98–1.70%), Mucoromycota (0.02–2.20%), Rozellomycota (0.32–1.39%), Aphelidiomycota (0.06–0.38%), Glomeromycota (0.04–0.25%), and Zoopagomycota (0.00–0.31%) (Fig. 3A). The top ten fungal genera in these samples were *Ganoderma* (0.16–20.81%), *Mortierella* (1.32–14.01%), *Gliocladiopsis* (0.16–14.91%), *Trichoderma* (2.74–9.77%), *Chaetomium* (2.10–7.27%), *Dictyochaeta* (0.92–9.76%), *Nadsonia* (0.34–11.29%), *Paracremonium* (0.18–4.08%), and *Solicoccozyma* (1.86–3.34%) (Fig. 3B).

### Fungal community composition comparison

**Fig. 4:**
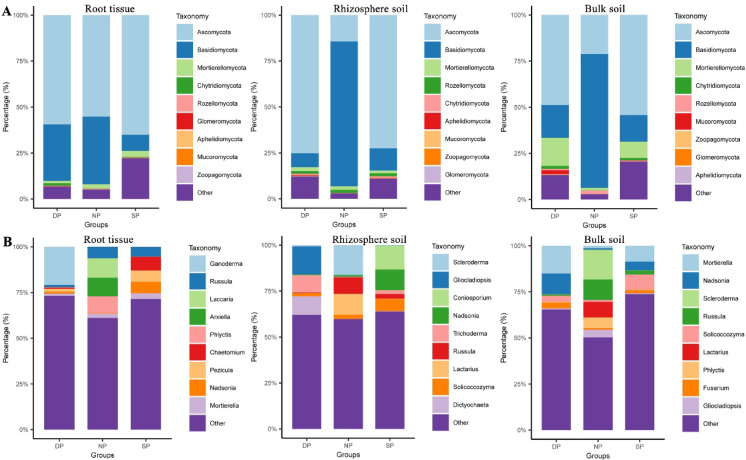
Top 10 dominant fungi relative abundance across different ecological niches. Comparison of the 10 most abundant fungi at the (**A**) phylum level and (**B**) genus level in root tissue, rhizosphere soil, and bulk soil. Dead, healthy, and diseased trees are abbreviated as DP, NP, and SP, respectively.

After identifying the fungal community composition in healthy, diseased, and dead *C. cathayensis* trees, we compared the fungal community composition of healthy, diseased, and dead *C. cathayensis* across the RT, RS, and BS groups to accurately analyze the presence of potentially pathogenic microorganisms (Fig. 4). In the RT group, the top 10 fungal phyla in the dead, diseased, and healthy trees were Ascomycota, Basidiomycota, Mortierellomycota, Chytridiomycota, Rozellomycota, Glomeromycota, Aphelidiomycota, Mucoromycota, and Zoopagomycota (Fig. 4A). Notably, the relative abundance of the top nine fungal phyla in diseased trees (77.83%) was lower than that in healthy (94.81%) and dead trees (93.32%) (Fig. 4A). The dominant fungal genera in these samples were *Ganoderma*, *Russula*, *Laccaria*, *Arxiella*, *Phlyctis*, *Chaetomium*, *Pezicula*, *Nadsonia*, and *Mortierella* (Fig. 4B). Interestingly, the total relative abundance and fungal composition of the top 9 fungal genera differed between healthy, diseased, and dead trees in the RT group (Fig. 4B).

In the RS group, the top ten fungal phyla in dead, diseased, and healthy trees were also dominated by Ascomycota, Basidiomycota, Mortierellomycota, Rozellomycota, Chytridiomycota, Aphelidiomycota, Mucoromycota, Zoopagomycota, and Glomeromycota (Fig. 4A). It is noteworthy that the total relative abundances and fungal compositions of the top 9 fungal genera in dead and diseased trees in the RS group were similar, but significantly different from those in healthy trees (Fig. 4A). Furthermore, the fungal community compositions in healthy, diseased, and dead trees in the RS group differed in terms of the top 9 fungal genera (Fig. 4B).

A similar trend was also found in the BS group as well. The total relative abundances and fungal compositions of the top 9 fungal genera in dead and diseased trees in the BS group were similar but different from those in healthy trees (Fig. 4A). Additionally, there were significant differences in the total relative abundances and fungal compositions among healthy, diseased, and dead trees in the BS group (Fig. 4B).

### Variance of fungal communities

**Fig. 5:**
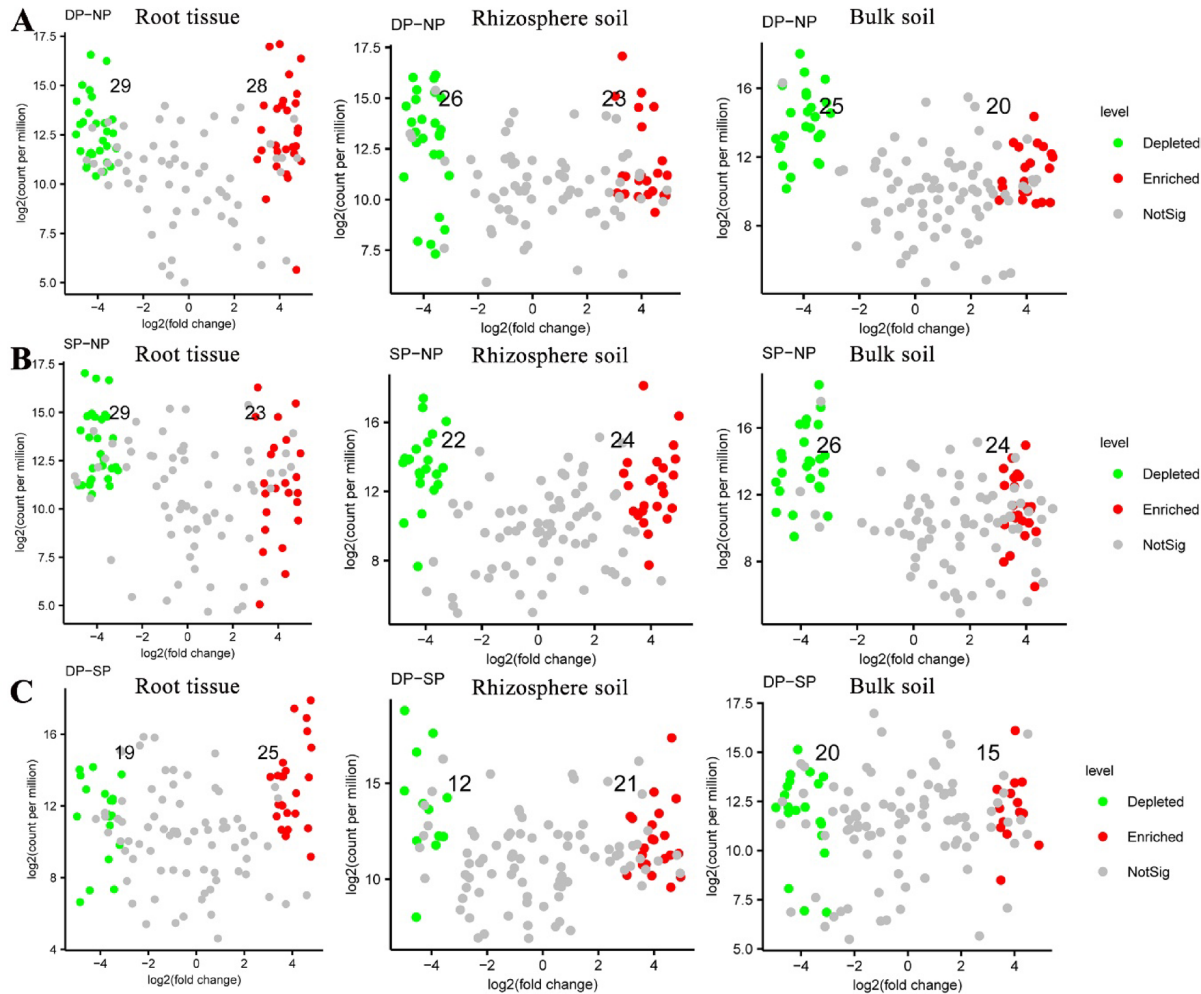
Variation in fungal abundance in root tissue, rhizosphere soil, and bulk soil samples. Changes in fungal communities in root tissue, rhizosphere soil, and bulk soil samples from (**A**) dead trees and (**B**) diseased trees compared to healthy trees, respectively. (**C**) Changes in fungal communities in root tissue, rhizosphere soil, and bulk soil samples from dead trees compared to diseased trees. fold-change > 2, *P* < 0.01. The numbers in the figure correspond to the quantities of green and red dots, which represent fungal ASVs that are significantly depleted and enriched, respectively. Dead, healthy, and diseased trees are abbreviated as DP, NP, and SP, respectively.

To reveal the effects of fungal communities on the health status of *C. cathayensis* trees, we compared the variation of fungal communities across healthy, diseased, and dead trees at the RT, RS, and BS levels (Fig. 5 and Supplementary Tables 1, 2, 3). In the root tissue group, the abundance of 29 fungal genera was significantly depleted, while 28 fungal genera were enriched in the DP group compared to the NP group (Fig. 5A and Supplementary Table 1) (*P* < 0.01). Similarly, 29 fungal genera were significantly depleted and 23 fungal genera were enriched in the SP group compared to the NP group (Fig. 5B and Supplementary Table 1) (*P* < 0.01). In addition, there were 19 depleted and 25 enriched fungal genera between the DP and SP groups (Fig. 5C and Supplementary Table 1) (*P* < 0.01).

In the rhizosphere soil group, the abundance of 26 fungal genera was depleted, and 23 fungal genera were enriched in the DP group compared to the NP group (Fig. 5A and Supplementary Table 2). In the SP group, the abundance of 22 fungal genera was significantly depleted, and 24 fungal genera were enriched compared to the NP group (Fig. 5B and Supplementary Table 2). Additionally, 12 fungal genera were depleted, and 21 fungal genera were enriched between the DP and SP groups (Fig. 5C and Supplementary Table 2) (*P* < 0.01).

In the bulk soil group, 25 fungal genera were depleted, and 20 fungal genera were significantly enriched in the DP group compared to the NP group (Fig. 5A and Supplementary Table 3). In the SP group, 26 fungal genera were depleted, and 24 fungal genera were enriched compared to the NP group (Fig. 5B and Supplementary Table 3). Similarly, there were 20 depleted and 15 enriched fungal genera between the DP and SP groups (Fig. 5C and Supplementary Table 3) (*P* < 0.01).

**Fig. 6:**
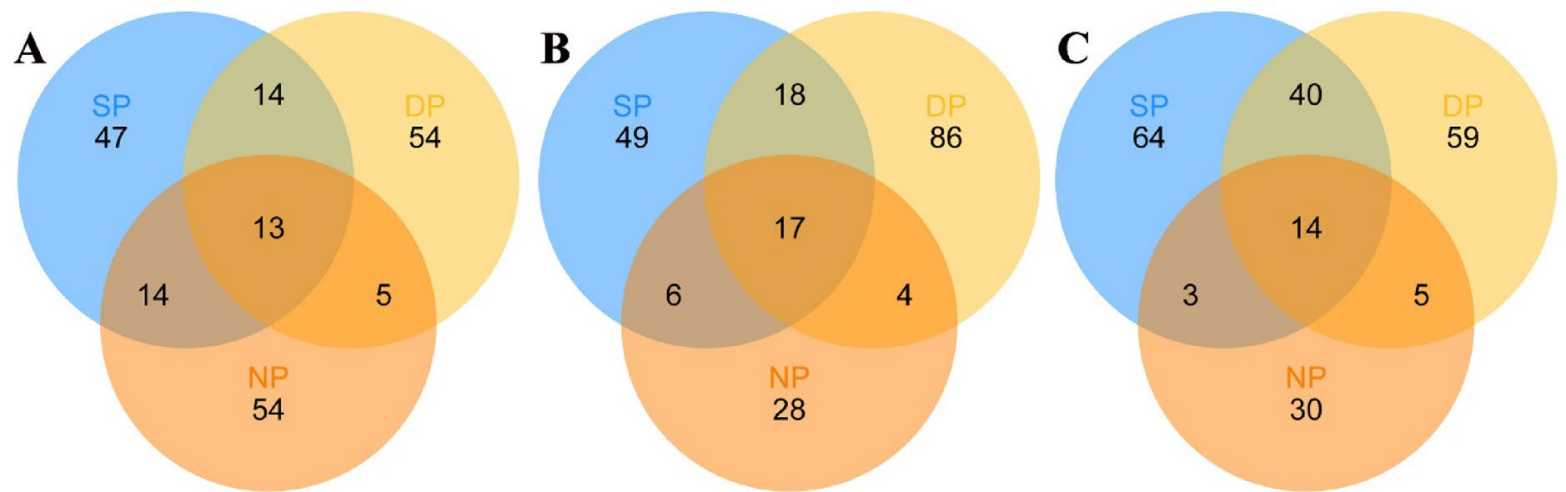
The common fungi in (**A**) root tissue, (**B**) rhizosphere soil, and (**C**) bulk soil of different tree groups shown in Venn diagrams. The numbers in the figure represent the number of fungal ASVs. DP, NP, and SP represent dead, healthy, and diseased trees, respectively.

Finally, We compared the common fungal communities of healthy, diseased, and dead *C. cathayensis* trees at the RT, RS, and BS levels (Fig. 6 and Supplementary Tables 4, 5, 6). In the root tissue group, a total of 13 fungal genera were shared between the NP, SP, and DP trees. Notably, 14 fungal genera were shared between the DP and SP trees (Fig. 6A and Supplementary Table 4). Likewise, a total of 17 fungal genera were shared between the NP, SP, and DP trees in the rhizosphere soil group, while 18 fungal genera were shared between the DP and SP trees (Fig. 6B and Supplementary Table 5). In the bulk soil group, 40 fungal genera were shared between the DP and SP trees. Additionally, 14 fungal genera were shared between the NP, SP, and DP trees (Fig. 6C and Supplementary Table 6).

### Microbiome Co-occurrence networks in NP, SP, and DP groups

**Fig. 7:**
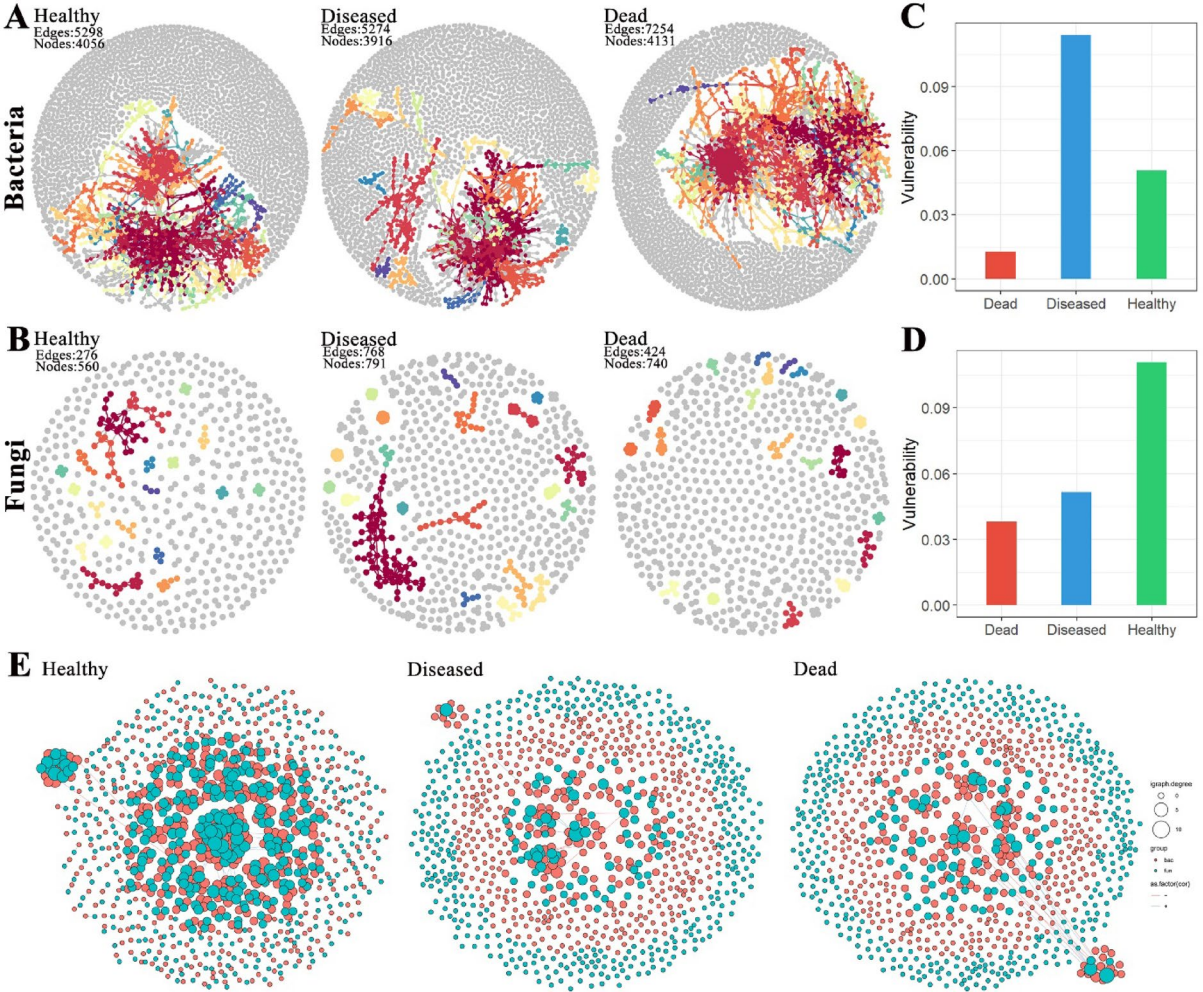
Ecological networks of microbial associations across different tree groups. (**A**) Bacterial network and (**B**) fungal network in the root soils of healthy, diseased, and dead *C. cathayensis* trees. Vulnerability of (**C**) bacterial networks and (**D**) fungal networks in the root soils of healthy, diseased, and dead *C. cathayensis* trees. (**E**) Bacterial-fungal cross-networks in the root soils of the healthy, diseased, and dead *C. cathayensis* trees. Each node represents an ASV and is colored according to its biological category, with pink denoting bacteria and blue denoting fungi. The size of each node corresponds to the degree of its respective ASV. Strong (*R* > 0.8) and statistically significant (*P* < 0.05) associations are indicated by connections between nodes.

To explore the differences in microbial network structures in the soil at the roots of healthy, diseased, and dead *C. cathayensis* trees, we analyzed previously published bacterial amplicon data^5^ and fungal amplicon data to calculate the topological characteristics of their co-occurrence networks. In the bacterial network, the soil from dead *C. cathayensis* trees contained 4131 nodes and 7254 edges, which was higher than the numbers found in the soil of healthy and diseased trees (Fig. 7A). For the fungal network, the soil from diseased *C. cathayensis* trees had 791 nodes and 768 edges, surpassing the soil of both healthy and dead trees in terms of network size (Fig. 7B). A network vulnerability analysis further revealed that the bacterial network in the soil of diseased *C. cathayensis* trees was more vulnerable, while the fungal network in the soil of healthy trees was more vulnerable (Fig. 7C,D). Additionally, we examined the cross-networks of bacteria and fungi in the root soils of healthy, diseased, and dead *C. cathayensis* trees. The soil from healthy trees possessed 1000 nodes and 992 edges, significantly more than those in the soil from diseased and dead trees (Table 1). Notably, the interaction between bacteria and fungi in the soil of healthy trees were predominantly positive, with very few negative correlation (Fig. 7E and Supplementary Data 1). In contrast, the soil of diseased and dead trees exhibited relatively independent bacterial and fungal networks, with fewer interactions and an approximately equal proportion of positive and negative correlations (Fig. 7E and Supplementary Data 2 and 3). The cross-network analysis indicated that a harmonious coexistence of bacteria and fungi in the root soils of *C. cathayensis* trees is crucial for preventing root rot disease.

**Table 1:** General characteristics of bacterial-fungal co-occurrence networks in healthy, diseased, and dead *C. cathayensis* trees.

Network feature	Healthy	Diseased	Dead
Number of nodes	1000	195	229
Number of edges	992	179	186
Diameter	22.11	17.73	20.79
Number of clusters	166	38	57
Average path length	9.03	7.16	8.36
Average degree	1.98	1.84	1.62
Centralization betweenness	0.055	0.139	0.136
Centralization degree	0.006	0.024	0.020
Centralization closeness	NA	NA	NA

### Distinction of the dominant fungal genera

**Fig. 8:**
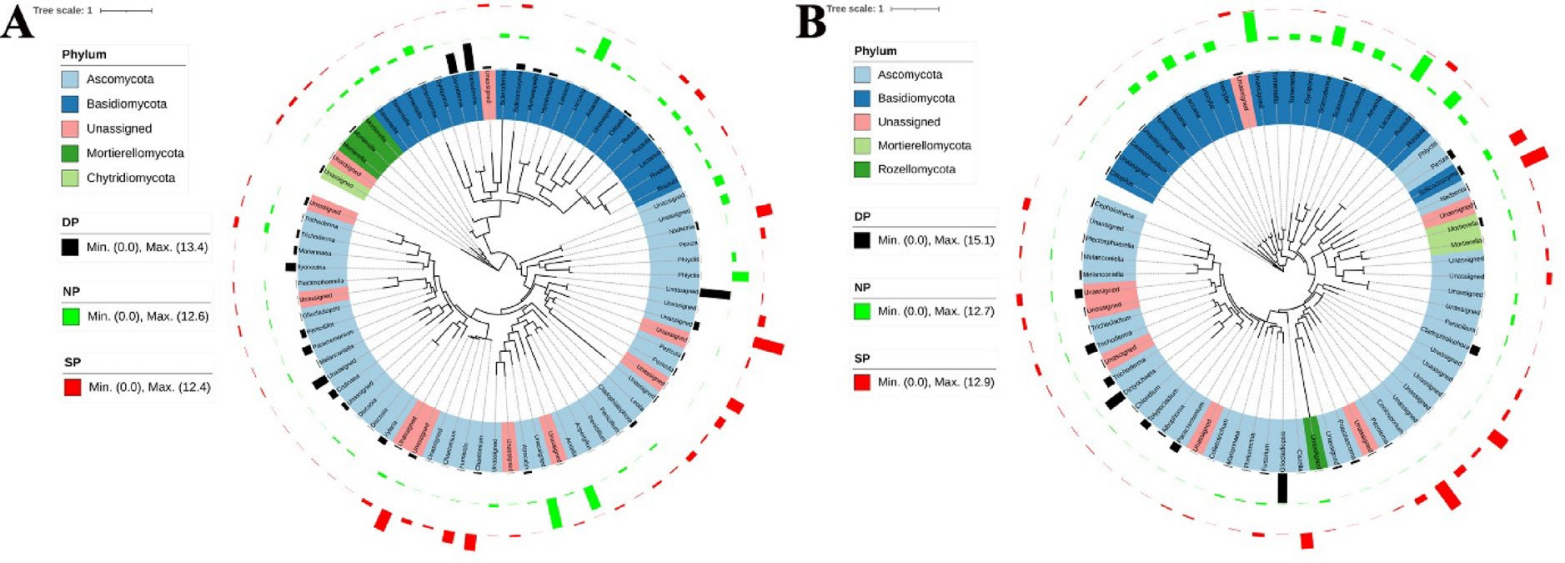
Taxonomic tree showing the relative abundance of the top 70 dominant fungi in (**A**) root tissue and (**B**) rhizosphere soil across different tree groups. Phyla within the tree are indicated by color ranges. The relative abundance of each ASV in dead, healthy, and diseased trees is represented by black, green, and red colored bars, respectively. iTOL was used to draw the taxonomic dendrogram. Dead, healthy, and diseased trees are abbreviated as DP, NP, and SP, respectively.

To provide a comprehensive overview of the dominant fungal genera in dead, healthy, and diseased *C. cathayensis* trees, we present the top 70 dominant fungal genera in phylogenetic trees at the RT and RS group levels (Fig. 8). In the RT group, the dominant fungal genera of dead *C. cathayensis* trees included *Ganoderma*, *Hymenopellis*, *Xylaria*, *Codinaea*, *Paracremonium*, *Penicillifer*, *Ilyonectria*, and *Mariannaea*. In contrast, the dominant fungal genera in diseased *C. cathayensis* trees in the RT group were *Nadsonia*, *Pezicula*, *Leotia*, *Humicola*, and *Chaetomium*. Healthy *C. cathayensis* trees in the RT group were rich in *Tomentella*, *Scleroderma*, *Laccaria*, *Amanita*, *Phlyctis*, and *Arxiella* (Fig. 8A).

In the RS group, the dominant fungal genera in the soil of dead *C. cathayensis* trees included *Peziza*, *Cladophialophora*, *Gliocladiopsis*, *Paracremonium*, *Dictyochaeta*, *Tolypociadium*, and *Trichoderma*. Similarly, diseased *C. cathayensis* trees of the RS group were rich in *Solicoccozyma*, *Nadsonia*, *Coniosporium*, and *Cistella*. In the case of healthy *C. cathayensis* trees in the RS group, *Hymenogaster*, *Laccaria*, *Inocybe*, *Scleroderma*, *Lactarius*, and *Russula* were the dominant fungal genera (Fig. 8B).

## Discussion

Root rot is a common soil-borne plant disease that seriously affects crop production worldwide^7^. In recent years, root rot in *C. cathayensis* has devastated the *C. cathayensis* industry, severely affecting production and local farmers’ income. However, the pathogen responsible for this disease remains unknown. Previous studies utilizing *16 S rRNA* amplicon sequencing identified imbalances in the root microbiota, particularly the reduction of certain probiotics, but did not uncover any potential pathogens^5^. To gain further insights into the pathogenesis of *C. cathayensis* root rot disease, we employed ITS1 amplicon sequencing to compare the fungal community composition in diseased, dead, and healthy *C. cathayensis* trees. The results revealed no significant differences in fungal richness and Shannon indices between diseased, dead, and healthy *C. cathayensis* in the RT and RS groups, indicating that there were no major changes in fungal richness or evenness across these conditions (Fig. 1A,B). However, in the BS group, we observed significant differences in fungal richness and evenness between diseased, dead, and healthy *C. cathayensis* trees (Fig. 1C). In addition, our results also showed that Ascomycota and Basidiomycota were the two dominant fungal phyla in the root tissue, rhizosphere soil, and bulk soil of *C. cathayensis* trees, accounting for approximately 93.63% of the total fungal community (Fig. 4A). This is consistent with previous studies, as these two phyla are the largest terrestrial fungal groups and their closest sister taxa^17,27^. Furthermore, in the RT and RS groups, the proportion of Ascomycota was higher in dead and diseased trees compared to healthy trees (Fig. 4A). In particular, Basidiomycota is dominant in RS and BS groups of healthy trees. However, in diseased and dead trees, the relative abundance of Ascomycota in the RS and BS groups was significantly higher than that of Basidiomycota (Fig. 4A). Interestingly, some Ascomycota fungi are known plant pathogens^24^, suggesting that the shift in fungal community composition could be linked to the onset of *C. cathayensis* root rot. This aligns with earlier reports^5,12,14,15,28,29^ that disturbances in fungal species balance can lead to plant diseases.

When analyzing the dominant microbiota in the RT and RS of diseased, dead, and healthy trees, we unexpectedly identified the dominant fungi *Xylaria* and *Ilyonectria* in the RT of dead trees, which were absent in healthy trees (Fig. 8A). *Condinaea* and *Gliocladiopsis* were predominantly present in dead trees, with less abundance in diseased and healthy trees (Fig. 8A and B). These findings are consistent with studies suggesting that *Xylaria* can cause root-related diseases, such as black root rot in apples and taproot decline in soybeans^30^. Additionally, *Codinaea fertilis* has been implicated in root rot in white clover^31^, while *Ilyonectria* is known to cause root rot and rust in ginseng^32^. *Gliocladiopsis* has also been linked to root rot in avocado^33^. Taken together, these results suggest that *Xylaria*, *Condinaea*, *Ilyonectria*, and *Gliocladiopsis* may be involved in the development of *C. cathayensis* root rot, marking the first report of potential pathogens in this context.

Furthermore, *Chaetomium* was abundant in the RT of diseased trees, but less so in healthy and dead trees (Fig. 8A). *Chaetomium globosum* is known to control a wide range of plant pathogens^34^, suggesting that *Chaetomium* may play a biocontrol role in *C. cathayensis* trees. Similarly, *Trichoderma*, which was abundant in the rhizosphere soil of dead and diseased trees, but less so in healthy trees (Fig. 8B), is an important biocontrol fungus that helps protect plants from disease^35^. These findings suggest that both *Chaetomium* and *Trichoderma* have potential as biocontrol agents. Their inhibitory activity against the candidate pathogen should be further validated through in vitro dual-culture tests in future studies.

Finally, the dominant fungi identified in the root tissues or rhizosphere soil of healthy trees included *Laccaria*, *Amanita*, *Inocybe*, *Lactarius*, and *Russula* (Fig. 8A,B), all of which belong to Basidiomycota. The presence of *L. ochropurpurea* has been shown to enhance pathogen resistance in chestnut plantations (*Castanea dentata*)^36^. *Inocybe*, *Lactarius*, *Russula*, and *Amanita* are the dominant fungal species in *C. illinoinensis* trees^37^. These fungi are likely to serve as beneficial probiotics for *C. cathayensis* trees.

## Supplementary Information

Below is the link to the electronic supplementary material.

Supplementary Material 1

Supplementary Material 2

Supplementary Material 3

Supplementary Material 4

Supplementary Material 5

Supplementary Material 6

## Data Availability

The clean data for this study can be obtained from the Genome Sequence Archive in National Genomics Data Centre^38^ under accession number CRA018861 at https://ngdc.cncb.ac.cn/gsa.

## References

[1] Shen, J., Li, X., Chen, X., Huang, X. & Jin, S. The complete Chloroplast genome of *Carya cathayensis* and phylogenetic analysis. *Genes (Basel)*. **13** (2), 369. 10.3390/genes13020369 (2022). 35205413 10.3390/genes13020369 PMC8871582

[2] Huang, J. et al. The mechanism of high contents of oil and oleic acid revealed by transcriptomic and lipidomic analysis during embryogenesis in *Carya cathayensis* Sarg. *BMC Genom.* **17**, 113. 10.1186/s12864-016-2434-7 (2016). 10.1186/s12864-016-2434-7 PMC4755018 26878846

[3] Li, Y. et al. Comparative analysis of aroma profiles in walnut, Pecan and hickory nuts during the roasting process using E-nose, HS-SPME-GC-MS, and HS-GC-IMS. *LWT* **210**, 116810. 10.1016/j.lwt.2024.116810 (2024).

[4] Xing, J. et al. A new insight into spacing patterns of soil bacterial Microbiome induced by root rot of *Carya cathayensis*. *Appl. Soil. Ecol.* **174**, 104416. 10.1016/j.apsoil.2022.104416 (2022).

[5] Bai, X. H. et al. Bacterial Microbiome differences between the roots of diseased and healthy Chinese hickory (*Carya cathayensis*) trees. *J. Microbiol. Biotechnol.* **33** (10), 1–11. 10.4014/jmb.2304.04054 (2023). 37528558 10.4014/jmb.2304.04054 PMC10619558

[6] Fang, W. et al. Deciphering differences in microbial community characteristics and main factors between healthy and root rot-infected *Carya cathayensis* rhizosphere soils. *Front. Microbiol.* **15**, 1448675. 10.3389/fmicb.2024.1448675 (2024). 39588107 10.3389/fmicb.2024.1448675 PMC11586369

[7] Williamson-Benavides, B. A. & Dhingra, A. Understanding root rot disease in agricultural crops. *Horticulturae* **7** (2), 33. 10.3390/horticulturae7020033 (2021).

[8] Liu, Y. et al. Distribution and pathogenicity of *Fusarium* species associated with soybean root rot in Northeast China. *Plant. Pathol. J.* **39** (6), 575–583. 10.5423/PPJ.OA.06.2023.0086 (2023). 38081317 10.5423/PPJ.OA.06.2023.0086 PMC10721389

[9] Chang, X. et al. Identification of *Fusarium* species associated with soybean root rot in Sichuan Province, China. *Eur. J. Plant. Pathol.* **151**, 563–577. 10.1007/s10658-017-1410-7 (2018).

[10] Yan, H. & Nelson, B. Effect of temperature on *Fusarium Solani* and *F. tricinctum* growth and disease development in soybean. *Can. J. Plant Pathol.* **42** (4), 527–537. 10.1080/07060661.2020.1745893 (2020).

[11] Zhang, C. Q., Liu, Y. H. & Xu, B. C. First report of *Fusarium* root rot on Chinese hickory (*Carya cathayensis*) caused by *Fusarium oxysporum* in China. *Plant. Dis.* **99** (9), 1284. 10.1094/PDIS-01-15-0073-PDN (2015).

[12] Chen, T. et al. A plant genetic network for preventing dysbiosis in the phyllosphere. *Nature* **580** (7805), 653–657. 10.1038/s41586-020-2185-0 (2020). 32350464 10.1038/s41586-020-2185-0 PMC7197412

[13] Huyben, D. et al. Dietary live yeast and increased water temperature influence the gut microbiota of rainbow trout. *J. Appl. Microbiol.* **124** (6), 1377–1392. 10.1111/jam.13738 (2018). 29464844 10.1111/jam.13738

[14] Paasch, B. C. & He, S. Y. Toward Understanding microbiota homeostasis in the plant Kingdom. *PLoS Pathog*. **17** (4), e1009472. 10.1371/journal.ppat.1009472 (2021). 33886694 10.1371/journal.ppat.1009472 PMC8061798

[15] Bodah, E. T. Root rot diseases in plants: A review of common causal agents and management strategies. *Agric. Res. Technol.* **5** (3), 555661. 10.19080/ARTOAJ.2017.05.555661 (2017).

[16] Berbee, M. L. The phylogeny of plant and animal pathogens in the Ascomycota. *Physiol. Molecul Plant. Pathol.* **59** (4), 165–187. 10.1006/pmpp.2001.0355 (2001).

[17] Arnault, G., Mony, C. & Vandenkoornhuyse, P. Plant microbiota dysbiosis and the Anna karenina principle. *Trends Plant. Sci.* **28** (1), 18–30. 10.1016/j.tplants.2022.08.012 (2023). 36127241 10.1016/j.tplants.2022.08.012

[18] Koranda, M. et al. Microbial processes and community composition in the rhizosphere of European beech - The influence of plant C exudates. *Soil. Biol. Biochem.* **43** (3), 551–558. 10.1016/j.soilbio.2010.11.022 (2011). 21412402 10.1016/j.soilbio.2010.11.022 PMC3032887

[19] Xin, Z. & Chen, J. A high throughput DNA extraction method with high yield and quality. *Plant. Methods*. **8** (1), 26. 10.1186/1746-4811-8-26 (2012). 22839646 10.1186/1746-4811-8-26 PMC3441248

[20] Toju, H., Tanabe, A. S., Yamamoto, S. & Sato, H. High-coverage ITS primers for the DNA-based identification of ascomycetes and basidiomycetes in environmental samples. *PLoS One*. **7** (7), e40863. 10.1371/journal.pone.0040863 (2012). 22808280 10.1371/journal.pone.0040863 PMC3395698

[21] X. Liu, Y. et al. EasyAmplicon: an easy-to-use, open-source, reproducible, and community-based pipeline for amplicon data analysis in Microbiome research. *iMeta* **2** (1), e83. 10.1002/imt2.83 (2023). 38868346 10.1002/imt2.83 PMC10989771

[22] Nilsson, R. H. et al. The UNITE database for molecular identification of fungi: handling dark taxa and parallel taxonomic classifications. *Nucleic Acids Res.*10.1093/nar/gky1022 (2019). 30371820 10.1093/nar/gky1022 PMC6324048

[23] Edgar, R. C. Search and clustering orders of magnitude faster than BLAST. *Bioinformatics* **26** (19), 2460–2461. 10.1093/bioinformatics/btq461 (2010). 20709691 10.1093/bioinformatics/btq461

[24] Robinson, M. D., McCarthy, D. J. & Smyth, G. K. EdgeR: a bioconductor package for differential expression analysis of digital gene expression data. *Bioinformatics* **26** (1), 139–140. 10.1093/bioinformatics/btp616 (2010). 19910308 10.1093/bioinformatics/btp616 PMC2796818

[25] Wen, T. et al. GgClusterNet: an R package for Microbiome network analysis and modularity-based multiple network layouts. *Imeta* **1** (3), e32. 10.1002/imt2.32 (2022). 38868720 10.1002/imt2.32 PMC10989811

[26] McKnight, D. T. et al. MicroDecon: A highly accurate read-subtraction tool for the post-sequencing removal of contamination in metabarcoding studies. *Environ. DNA*. **1**, 14–25. 10.1002/edn3.11 (2019).

[27] Egidi, E. et al. A few Ascomycota taxa dominate soil fungal communities worldwide. *Nat. Commun.* **10** (1), 2369. 10.1038/s41467-019-10373-z (2019). 31147554 10.1038/s41467-019-10373-z PMC6542806

[28] Kong, H. G., Ham, H., Lee, M. H., Park, D. S. & Lee, Y. H. Microbial community dysbiosis and functional gene content changes in Apple flowers due to fire blight. *Plant. Pathol. J.* **37** (4), 404–412. 10.5423/PPJ.NT.05.2021.0072 (2021). 34365752 10.5423/PPJ.NT.05.2021.0072 PMC8357563

[29] Lee, S. M., Kong, H. G., Song, G. C. & Ryu, C. M. Disruption of firmicutes and actinobacteria abundance in tomato rhizosphere causes the incidence of bacterial wilt disease. *ISME J.* **15** (1), 330–347. 10.1038/s41396-020-00785-x (2021). 33028974 10.1038/s41396-020-00785-x PMC7852523

[30] Garcia-Aroca, T. et al. *Xylaria necrophora*, sp. nov., is an emerging root-associated pathogen responsible for taproot decline of soybean in the Southern united States. *Mycologia* **113** (2), 326–347. 10.1080/00275514.2020.1846965 (2021). 33555993 10.1080/00275514.2020.1846965

[31] Menzies, S. A. Root rot of clover caused by *Codinaea fertilis*. *New. Z. J. Agric. Res.* **16** (2), 239–245. 10.1080/00288233.1973.10421141 (1973).

[32] Farh, M. E., Kim, Y. J., Kim, Y. J. & Yang, D. C. Cylindrocarpon destructans/Ilyonectria radicicola-species complex: causative agent of ginseng root-rot disease and Rusty symptoms. *J. Ginseng Res.* **42** (1), 9–15. 10.1016/j.jgr.2017.01.004 (2018). 29348716 10.1016/j.jgr.2017.01.004 PMC5766697

[33] Parkinson, L. E., Shivas, R. G. & Dann, E. K. Novel species of *Gliocladiopsis* (Nectriaceae, Hypocreales, Ascomycota) from avocado roots (*Persea americana*) in Australia. *Mycoscience* **58** (2), 95–102. 10.1016/j.myc.2016.10.004 (2017).

[34] Ashwini, C. A review on *Chaetomium globosum* is versatile weapons for various plant pathogens. *J. Pharmacogn Phytochem*. **8** (2), 946–949 (2019).

[35] Vinale, F. et al. Trichoderma–plant–pathogen interactions. *Soil. Biol. Biochem.* **40** (1), 1–10. 10.1016/j.soilbio.2007.07.002 (2008).

[36] Ritzi, M. V., Russell, S. D., Aime, M. C. & McNickle, G. G. First report of ectomycorrhizal fungus *Laccaria ochropurpurea*, associated with *Castanea dentata* (American chestnut) roots in a mixed species plantation. *Plant. Health Prog*. **20**, 140–141. 10.1094/PHP-01-19-0008-BR (2019).

[37] Ge, Z. W., Brenneman, T., Bonito, G. & Smith, M. E. Soil pH and mineral nutrients strongly influence truffles and other ectomycorrhizal fungi associated with commercial pecans (*Carya illinoinensis*). *Plant. Soil.* **418**, 493–505. 10.1007/s11104-017-3312-z (2017).

[38] Chen, T. et al. The genome sequence archive family: toward explosive data growth and diverse data types. *Genomics Proteom. Bioinforma*. **19** (4), 578–583. 10.1016/j.gpb.2021.08.001 (2021). 10.1016/j.gpb.2021.08.001 PMC9039563 34400360

