# Supplementary figures and images for: Analysing fungal microbiome differences between the roots of healthy and diseased Chinese hickory (Carya cathayensis) trees
Source: Sci Rep. 2025 Dec 17;15:44018. doi: 10.1038/s41598-025-27645-y (PMC12712095; doi:10.1038/s41598-025-27645-y)

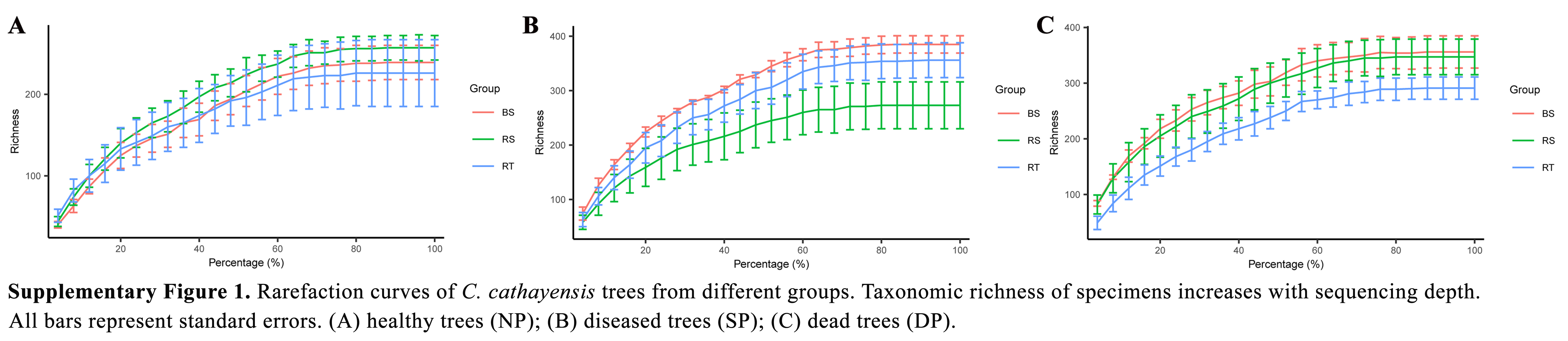

Supplement: Supplementary file 6 — Supplementary Material 6 [file 41598_2025_27645_MOESM6_ESM.jpg]
